# Clinical Efficacy of SPARC-Modified Mesenchymal Stem Cells for the Treatment of Dog Skin Wounds

**DOI:** 10.3390/vetsci13030222

**Published:** 2026-02-26

**Authors:** Hong-Kai Tian, Ba-Lun Li, Jia-Qi Gao, Dong-Yao Han, Nikita Merzlikin, Chen-Chen Li, Zi-Xi Ling, Zeng-Yu Zhang, Wen-Long Zhu, Jian-Qi Dai, Lydmila Gerunova, Le-Xi Gao, Na Li, Jin-Lian Hua

**Affiliations:** 1 College of Veterinary Medicine, Shaanxi Centre of Stem Cells Engineering & Technology, Northwest A&F University, Xianyang 712100, China; 2022055543@nwafu.edu.cn (H.-K.T.); libalun@nwafu.edu.cn (B.-L.L.); gaojq@nwafu.edu.cn (J.-Q.G.); han1001@nwsuaf.edu.cn (D.-Y.H.); merzlickin.nikita2015@yandex.ru (N.M.); lcc19980902@163.com (C.-C.L.); 16602914351@163.com (Z.-X.L.); 2018010993@nwafu.edu.cn (Z.-Y.Z.); 2019010901@nwafu.edu.cn (W.-L.Z.); daijianqi@nwafu.edu.cn (J.-Q.D.); gaolexi@nwafu.edu.cn (L.-X.G.); lina2017@nwafu.edu.cn (N.L.); 2 Faculty of Veterinary Medicine, Omsk State Agrarian University, Omsk 644122, Russia; lk.gerunova@omgau.org

**Keywords:** secreted protein acidic and rich in cysteine (SPARC), adipose-derived mesenchymal stem cells (ADSCs), skin wound, diabetes, repair

## Abstract

The number of pet dogs is increasing, and the number of working dogs (e.g., guide dogs, police dogs) is also gradually increasing. Skin wounds are a common clinical problem in dogs and tend to be more common in the clinic as mechanical wounds. The healing process of skin wounds is often influenced by a variety of factors, including infection, nutritional status, and immune response, while wound healing is more difficult in dogs with diabetes or aging dogs. Mesenchymal stem cells (MSCs) play an important role in skin healing and regeneration with their multidirectional differentiation potential and immunomodulatory function. However, the application of MSCs alone for the treatment of skin wounds may have certain limitations, such as low cell survival and a lack of localization. Therefore, it is important to find methods that can enhance the therapeutic effect of MSCs. Secreted protein acidic and rich in cysteine (SPARC), an extracellular matrix protein widely involved in regulating biological processes such as cell proliferation, migration, and matrix production, may enhance the efficacy of MSCs in skin wound healing. This study aims to systematically evaluate the therapeutic efficacy of SPARC-overexpressing adipose-derived mesenchymal stem cells (ADSCs) in promoting skin wound healing by establishing wound models in normal, diabetic, and aged mice and dogs, thereby validating their potential under diverse physiological and pathological conditions. For in vitro validation, we used hydrogen peroxide (H_2_O_2_) to induce Human Umbilical Vein Endothelial Cell (HUVEC) and Human Keratinocyte Cell (HaCaT) injury. All animals were randomly assigned to six experimental groups as follows: (1) Model group: Untreated wound (negative control); (2) HY group: Hydrogel alone (vehicle control); (3) Con group: Control-ADSCs (cell control); (4) Con-Exo&HY group: Control-ADSC exosomes in hydrogel; (5) SPARC group: oe-SPARC-ADSCs (treatment); (6) SPARC-Exo&HY group: oe-SPARC-ADSC exosomes in hydrogel (treatment). Separately, HUVEC and HaCaT cells were assigned to four experimental conditions: a blank control group, a model group, a control-ADSC-treated group, and an oe-SPARC-ADSC-treated group. ADSCs modified by SPARC significantly promoted re-epithelialization integrity, collagen deposition, inflammation reduction, angiogenesis, and hair follicle regeneration during wound healing in dog skin. HUVEC and HaCaT cells proliferated after adding oe-SPARC-ADSCs cell supernatant. Meanwhile, quantitative proteomic sequencing data analysis showed that SPARC could promote skin wound healing by enhancing cell adhesion, hyaluronic acid binding, and vascular smooth muscle contraction of ADSCs. Both in vitro cellular assays and in vivo wound-healing models suggest that the combination of SPARC and ADSCs for the treatment of skin wounds has broad application prospects.

## Simple Summary

Skin wounds are a common health issue in dogs, particularly for aging or diabetic animals, where healing can be challenging. Mesenchymal stem cells are promising for treatment but face limitations like poor survival at the wound site. This study explores a new strategy by using adipose-derived stem cells (ADSCs) that are engineered to produce high levels of a protein called SPARC. In a dog wound model, these modified cells significantly improved healing by enhancing skin regrowth, collagen production, new blood vessel formation, and hair follicle regeneration while reducing inflammation. Laboratory tests on human skin and blood vessel cells also showed that the secretions from these SPARC-enhanced stem cells promoted cell growth. Overall, combining SPARC with ADSCs presents a highly effective and promising approach for treating difficult skin wounds in dogs.

## 1. Introduction

The skin is the largest organ in mammals. It has many functions, such as the regulation of the body’s fluid and temperature, sensation, synthesis of vitamin D, and acting as a barrier between the body and the environment [1]. The skin is divided into two layers, the epidermis and the dermis. The epidermis is the outermost complex flat epithelium surface of the skin, which can be subdivided into two parts: the stratum corneum and the germinal layer. The dermis is a dense connective tissue with many elastic and collagen fibers that are elastic and tough. The dermis is thicker than the epidermis and is rich in blood vessels and nerves. Below the skin, there is subcutaneous tissue, which is loose connective tissue with a large number of fat cells. Because the skin is located on the body’s surface, it is susceptible to external injury and the effects of aging or reduced blood circulation [2,3,4]. In clinical treatment, skin wounds caused by trauma are the most common [5]. However, in addition to this, animals with diabetes or aging have difficulty in healing wounds due to the presence of underlying diseases or endocrine, metabolic factors, and variables such as gait and stance, which likely contribute to dermatological injuries and delayed wound healing, often leading to a series of complications such as ulcers. Repair after skin wound is an important physiological process to maintain the integrity of the skin, particularly in the face of specific clinical challenges such as granulomas, pressure point ulcers, or pedal wounds, and is a complex biological process, which generally includes four stages: coagulation and hemostasis, inflammation, cell proliferation, and tissue remodeling [6]. Traditional methods of treating skin wounds include dressing bandages, negative pressure wound therapy, autologous skin grafting, and hyperbaric wound therapy [7,8,9], which have limitations such as high cost, inability to remove inactivated tissues on time, surgical debridement, and oxygen toxicity. While effective in many cases, these approaches can have limitations, which has spurred the development of novel, non-traditional strategies such as cellular therapies, including those utilizing stem cells.

In recent years, cell therapy has come into the limelight as a possible treatment. Mesenchymal stem cells (MSCs), as a type of pluripotent stem cells, can differentiate into chondrocytes, myoblasts, and adipocytes. MSCs are present in most tissues, mainly in bone marrow and adipose tissues [10]. MSCs have been used in disease models to control multiple steps of wound healing and regenerative processes [11,12]. Adipose-derived mesenchymal stem cells (ADSCs) were selected for our subsequent experiments because of their easy accessibility. Secreted protein acidic and rich in cysteine (SPARC), also known as BM-40 or osteonectin, is a stromal cell (extracellular matrix-associated) protein [13]. Several studies have demonstrated that SPARC can optimize the regenerative environment and improve cell regeneration from different perspectives (metabolism, tissue repair, oxidation, inflammation, cancer, etc.) [14]. SPARC plays multiple roles in wound healing, promoting cell migration, proliferation, and matrix remodeling, which can contribute to accelerating wound healing. SPARC can also influence the proliferation and differentiation of stromal cells and promote new matrix synthesis and remodeling, which helps to form tough wound tissue [15]. It has been found that the beneficial effects of SPARC on wound healing may be related to its regulation of cell–matrix interactions, promotion of neovascularization, modulation of apoptosis, and other multiple mechanisms [16,17]. Therefore, SPARC plays an important role in the wound-healing process and helps to improve the speed and quality of wound healing.

**Figure 1:**
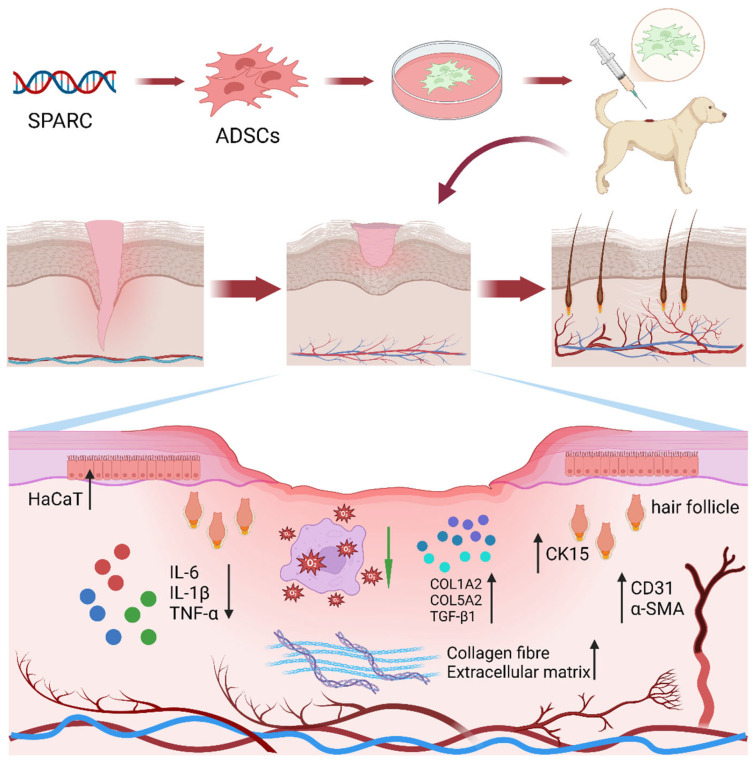
Patterns of SPARC-modified ADSCs that promote wound healing in the skin of the dog.

In summary, while SPARC holds promise in regenerative medicine, its distinct role in wound healing remains underexplored. Therefore, we hypothesized that SPARC overexpression in ADSCs would enhance their paracrine function and consequently accelerate wound closure and tissue remodeling across multiple physiological and pathological conditions. To test this hypothesis, we systematically investigated the therapeutic efficacy of SPARC-overexpressing ADSCs in three well-established animal models: normal, diabetic, and aged mice and dogs (Figure 1).

## 2. Materials and Methods

### 2.1. Animal and Skin Wound Modeling

All experimental protocols were conducted according to the guidelines established by the Chinese national standard GB/T35892-2018 (Ethical Review Guidelines for Laboratory Animal Welfare) [18].

Male ICR (Institute of Cancer Research) mice, 8 weeks old, were purchased from the Laboratory Animal Center of Chengdu Dashuo Laboratory Animal Co. (Chengdu, China). All animals were housed under laboratory conditions and acclimatized to the environment for at least one week at a temperature of 22 ± 2 °C, humidity of 50–60%, and provided with sufficient water and food. The mouse models of diabetes were established by intraperitoneal injection of streptozotocin (STZ) at 40 mg/kg for 4 days. Blood glucose levels were measured after one week to ensure the effectiveness of the modeling. The mouse models of aging were established by continuous daily subcutaneous injection of 150 mg/kg of D-galactose (D-gal), and after 6 weeks of modeling, the mice were seen to be slow-moving, with rough and sparse coats.

Six male crossbreed dogs, weighing about 6 kg, were obtained from the Experimental Animal Center of Northwest Agriculture and Forestry University (Xianyang, China). All experimental dogs were kept in a ventilated and clean environment and given adequate food and water. On day 7, model dogs with diabetes were established by a single intravenous injection of STZ at a dose rate of 35 mg/kg. On day 14, blood glucose levels were measured to determine diabetes modeling. Model dogs with aging were established by daily subcutaneous injection of 80 mg/kg D-gal, and slow movement, prolonged lying, and rough coat were found in the model dogs at week 8 of modeling.

### 2.2. Cell Isolation and Culture

We used the oe-SPARC-ADSCs cell line from a previously established cell line in the laboratory [19]. Cells were cultured in an incubator at 37 °C with 5% CO_2_ using α-MEM medium with 10% fetal bovine serum. When the cell density reached about 80%, the cells were digested with 0.25% trypsin [20].

### 2.3. Exosome Extraction

Cell supernatant for exosomes was extracted by centrifugation at 300× *g* for 10 min to remove dead cells. The collected supernatant was then centrifuged at 2000× *g* for 10 min to remove cellular debris. Carefully collect the supernatant, 10,000 *g*, centrifuge for 30 min, and remove larger stray proteins. Carefully collect the supernatant, 100,000 *g*, centrifuge for 60 min, discard the supernatant, resuspend the precipitate in phosphate buffer saline (PBS), 100,000 *g*, centrifuge for 60 min. Obtain the exosomes and resuspend them in an appropriate amount of PBS.

### 2.4. Giemsa Staining

Control-ADSCs and oe-SPARC-ADSCs were treated with H_2_O_2_ for 24 h, then the cells were exchanged for normal culture medium for 24 h. The supernatants of each group were discarded, and cells were fixed with 4% paraformaldehyde for 20 min at room temperature. Washed the cells three times with PBS and stained dropwise with Giemsa stain working solution for 15 min. After washing three more times with PBS, the cells were observed under an optical microscope according to the instructions provided with the Giemsa staining kit (Beyotime, Shanghai, China) [21].

### 2.5. Establishment of Skin Wound Model in Mice

Before performing the experiment, general anesthesia was induced to reduce pain in animals with intraperitoneal injection of tribromoethanol. The dorsal surface of the mice was shaved before surgery to minimize surgical site infection, and the surgical area was disinfected with 75% alcohol solution. Using a sterile scalpel, a circular wound of 1 cm in diameter was cut from the mice’s dorsal side. The trauma depth was meticulously controlled within the dermis to prevent any injury to the subcutaneous tissue beneath. Following the identification of the trauma, it was carefully rinsed with saline to eliminate blood and debris while maintaining a sterile environment around the wound. Mice were randomly divided into the following groups: (1) Model group: Untreated wound (negative control); (2) HY group: Hydrogel alone (vehicle control); (3) Con group: Control-ADSCs (cell control); (4) Con-Exo&HY group: Control-ADSC exosomes in hydrogel; (5) SPARC group: oe-SPARC-ADSCs (treatment); (6) SPARC-Exo&HY group: oe-SPARC-ADSC exosomes in hydrogel (treatment), with each group containing at least 3 mice to ensure statistical significance. Treatment was performed every 3 days using localized subcutaneous cell transplantation of the trauma. Wound healing was assessed on different days postoperatively (5, 10, and 15 days). The main parameters observed included wound shrinkage, healing time, and histopathologic examination. Wound shrinkage was calculated by measuring the diameter of the wound and the changes relative to the postoperative wound area [12].

### 2.6. Establishment of Skin Wound Model in Dogs

The dogs were fasted for 24 h before surgery. General anesthesia with intramuscular injection of xylazine hydrochloride. Physiological parameters such as respiration and heart rate were continuously monitored to ensure optimal anesthesia. After partially shaving and sterilizing the dog’s dorsum, six incisions with a diameter of 1.5 cm were created using a scalpel. These incisions were allocated to the following groups: (1) Model group: Untreated wound (negative control); (2) HY group: Hydrogel alone (vehicle control); (3) Con group: Control-ADSCs (cell control); (4) Con-Exo&HY group: Control-ADSC exosomes in hydrogel; (5) SPARC group: oe-SPARC-ADSCs (treatment); (6) SPARC-Exo&HY group: oe-SPARC-ADSC exosomes in hydrogel (treatment). The incision depth was controlled within the dermis to prevent damage to the underlying subcutaneous tissue. Postoperatively, the wound was cleaned with saline and maintained in a clean and dry condition. Treatment involving localized subcutaneous cell transplantation at the wound sites was conducted every three days, with regular observations of wound healing scheduled at 5, 10, and 15 days post-surgery. Daily Wound cleaning, dressing changes, and close monitoring of postoperative activity of the dogs were performed to ensure recovery.

### 2.7. Histopathology Examination

After 15 days of treatment, the dogs were euthanized for histopathological examination. The newly formed skin tissues were removed, rinsed with PBS, and fixed in 4% paraformaldehyde at 4 °C for 24 h. Following fixation, the tissues were gradually dehydrated, embedded in paraffin, and 3 μm-thick sections were prepared. Subsequently, hematoxylin-eosin (H&E) and Masson staining were performed, and the degree of re-epithelialization, inflammatory cell infiltration, and collagen deposition of the neonatal skin tissues were observed under an optical microscope [22].

### 2.8. Immunohistochemical Staining

Paraffin sections were placed in a thermostat at 60 °C and baked for 60 min, followed by dewaxing and hydration: xylene (10 min) → xylene (10 min) → anhydrous ethanol (5 min × 2 times) → 95% ethanol (5 min × 2) → 90% ethanol (5 min) → 85% ethanol (5 min) → 80% ethanol (5 min) → 75% ethanol (5 min). After the ethanol series, the sections were rinsed twice in distilled water, each for 5 min. Next, the sections were antigenically repaired with Tris-EDTA buffer at pH 9.0. The sections were microwaved for 15–20 min and allowed to cool at room temperature. Following cooling, the sections were washed three times with PBS. To eliminate endogenous peroxidase activity, the sections were treated with 3% hydrogen peroxide (H_2_O_2_) for 15 min. After this treatment, a blocking solution consisting of 10% fetal bovine serum was applied, and the sections were incubated at room temperature for 1 h to minimize nonspecific binding. Primary antibodies against the target proteins (IL-6, IL-1β, TNF-α, CD31, α-SMA) were added dropwise to the sections, which were covered and incubated overnight at 4 °C. The sections were then incubated with PBS for 1 h at room temperature. Subsequently, the sections were washed three times with PBS, and 100 μL or an appropriate amount of enhanced enzyme-labeled sheep anti-mouse/rabbit IgG polymer was added dropwise. The sections were incubated at 37 °C for 20 min, and then washed three more times with PBS. For visualization, the sections were subjected to DAB chromogenic solution, and hematoxylin staining solution was used to develop nuclear staining. Finally, the samples were dehydrated, sealed with neutral resin droplets, and analyzed under an optical microscope (Nikon Corporation, Tokyo, Japan) [23].

### 2.9. Immunofluorescence Staining

Paraffin sections were placed in a thermostat at 60 °C and baked for 60 min, followed by dewaxing and hydration: xylene 10 min (twice) → anhydrous ethanol (5 min × 2 times) → 95% ethanol (5 min × 2) → 90% ethanol (5 min) → 85% ethanol (5 min) → 80% ethanol (5 min) → 75% ethanol (5 min), after the ethanol series, section was rinsed twice in distilled water for 5 min. The sections were antigenically repaired with Tris-EDTA buffer at pH 9.0. The sections were microwaved for 15–20 min and allowed to cool at room temperature. Following three washes with PBS, the sections were incubated for 1 h at room temperature using 10% fetal bovine serum to block nonspecific binding. Subsequently, the blocking solution was removed, the primary antibody (CK15, PCNA) was added dropwise, and incubated overnight at 4 °C. After three washes with PBS, the fluorescent secondary antibody was added to the sample. The sections were incubated for 1 h, protected from light, and washed three times with PBS. Finally, dropwise anti-fluorescent attenuating sealer containing DAPI was added, and the sections were observed under a fluorescence microscope [24,25].

### 2.10. HUVEC and HaCaT Culture

HUVEC and HaCaT were provided by the Key Laboratory of Livestock Biology at Northwest A&F University. Human Umbilical Vein Endothelial Cells (HUVEC) and Human Keratinocyte Cells (HaCaT) were cultured in an incubator at 37 °C with 5% CO_2_ using Dulbecco’s modified Eagle medium (DMEM) containing 10% fetal bovine serum. When the cell density reached about 80%, the passages were digested with 0.25% trypsin. HUVEC and HaCaT were inoculated into 6-well, 48-well, and 96-well plates according to the different experimental requirements of each assay [26,27].

### 2.11. Cell Scratching Experiment

Five parallel reference lines were drawn on the back of the 6-well plate to facilitate subsequent photographic recording. HUVEC or HaCaT cells were inoculated evenly into the 6-well plate (pre-treated with H_2_O_2_ for 24 h), and when the cells were completely fused, a 200 μL tip perpendicular to the labeled horizontal line was used to make a cellular scratch by scraping off the cells with an equal width. Excess supernatant was removed, and PBS was added to wash the cells twice. HUVEC or HaCaT were divided into four groups: the NC group, the Model group, the Control-ADSCs group, and the SPARC-ADSCs group, respectively. Then, 2 mL of DMEM containing 2% serum was added to the first two groups, and 1 mL of Control-ADSCs supernatant and 1 mL of DMEM containing 2% serum were added to the third group. To the fourth group, 1 mL of SPARC-ADSCs supernatant and 1 mL of DMEM containing 2% serum were added.

Cell culture was performed, and the scratch width was photographed at 0 h, 12 h, and 24 h. Furthermore, the cell culture was incubated, and the scratch width was recorded at 0 h, 12 h, and 24 h. Finally, the cell culture was incubated with 2 mL of DMEM containing 2% serum and 2 mL of DMEM.

### 2.12. CCK-8 Cell Proliferation Assay

HUVEC or HaCaT cell suspensions (100 μL/well) were inoculated into 96-well plates, and divided into the NC group, Model group, Control-ADSCs group, and SPARC-ADSCs group, with 6 replicated wells in each group. After cell attachment, H_2_O_2_ was used to incubate groups Model, Control-ADSCs, and SPARC-ADSCs for 24 h, after which the medium was replaced. Then, 50 μL of Control-ADSCs supernatant and 50 μL of DMEM medium containing 10% serum were added to the Control-ADSCs group. Similarly, for the SPARC-ADSCs group, 50 μL of SPARC-ADSCs supernatant and 50 μL of DMEM medium containing 10% serum were added. To the Model group, 100 μL of normal DMEM medium containing 10% serum was added. Finally, 10 μL of CCK-8 solution was added to each well after 24 h of incubation. The cells were incubated in a cell culture incubator for 1 h. An enzyme marker was used to determine the absorbance value at 450 nm [28].

### 2.13. Angiogenesis Experiment

HUVEC were pre-divided into four groups: the NC group, Model group, Control-ADSCs group, and SPARC-ADSCs group. H_2_O_2_ incubation for 24 h in groups: Model, Control-ADSCs, SPARC-ADSCs. For the Control-ADSCs group, 50 μL of Control-ADSCs supernatant and 50 μL of DMEM medium containing 10% serum were used in DMEM medium. Similarly, for the SPARC-ADSCs group, 50 μL of SPARC-ADSCs supernatant and 50 μL of DMEM medium containing 10% serum were added, and 100 μL of normal DMEM medium containing 10% serum was added to the model group. Then, 50 μL liquid matrix gel refrigerated at 4 °C was inoculated into 96-well plates using a sterile pipette and placed in a cell incubator at 37 °C for 20 min to solidify the matrix gel. With three replicate wells in each group, 30,000 HUVEC were resuspended in normal media containing 10% FBS and seeded onto the solidified matrix gel. The tubule formation was examined and captured on camera using a microscope, 10 h after being put in the cell culture incubator at 37 °C [29].

### 2.14. Sequencing Analysis

The cells were divided into the Control-ADSCs group and oe-SRARC-ADSCs group. The supernatant cell specimens were collected and sent to Beijing Novozymes Technology Co. (Beijing, China). Data-independent acquisition (DIA) quantitative proteome sequencing was performed according to the company’s instructions. Potential target gene information was analyzed by Gene Ontology (GO) and Kyoto Encyclopedia of Genes and Genomes (KEGG) pathway using annotation databases. The pathway with *p* value < 0.05 was considered reliable.

### 2.15. Statistical Analysis

Data were collated and analyzed using GraphPadPrism9 software and tested for significant differences using *t*-test and one-way ANOVA. Significant differences are indicated as follows: * stands for *p* < 0.05, ** stands for *p* < 0.01, *** stands for *p* < 0.001. **** stands for *p* < 0.0001 and ns stands for no significant difference.

## 3. Results

### 3.1. Overexpression of SPARC Improves the Resistance of Mesenchymal Stem Cells, Endothelial, and Keratinocytes to Injury

**Figure 2:**
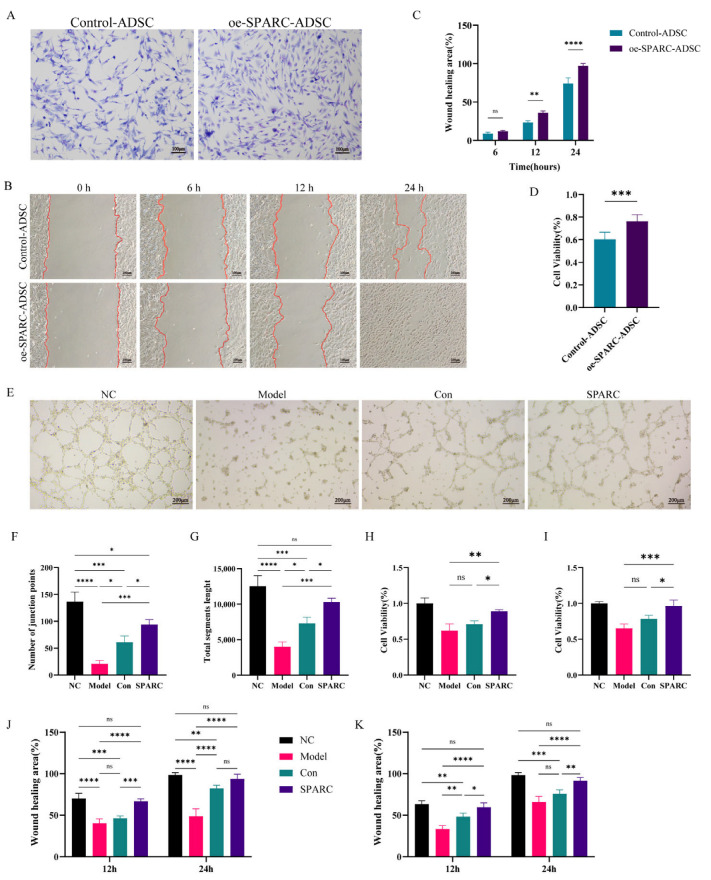
SPARC enhances the resistance of MSCs, endothelial, and keratinocytes to injury. (**A**) Giemsa staining; (**B**) Migration assay; (**C**) Quantification of the results of the migration assay; (**D**) Detection of cell proliferation by CCK-8 assay; (**E**) Tubule formation assay to detect the tube-forming ability of HUVEC cells after treatment with H_2_O_2_ and SPARC; (**F**) Number of nodes in the HUVEC vasculature formation assay; (**G**) Total vessel length of HUVEC angiogenesis assay; (**H**) HUVEC proliferation ability detected by CCK-8 assay; (**I**) HaCaT proliferation ability detected by CCK-8 assay; (**J**) Quantification of the results of the HUVEC scratch assay; (**K**) Quantification of the results of the HaCaT scratch assay. ns: Difference is not significant; *: *p* < 0.05; **: *p* < 0.01; ***: *p* < 0.001; ****: *p* < 0.0001.

To investigate the effects of overexpression of SPARC on different cellular resistance to injury, we treated ADSCs overexpressing SPARC with H_2_O_2_. Cell brightfield photographs with Giemsa staining showed that ADSCs overexpressing SPARC maintained a better morphology and cellular integrity after injury (Figure 2A and Figure S1a). Furthermore, scratch experiments showed that ADSCs migration in the wound surroundings might be enhanced by SPARC overexpression. (Figure 2B,C). ADSCs overexpressing SPARC had a significantly higher proliferative capacity than Control-ADSCs during injury, as detected by the CCK-8 method (Figure 2D).

HUVEC and HaCaT were used to simulate the wound-healing process in vitro. H_2_O_2_ was used to treat the two cell types independently, and an oe-SPARC-ADSC supernatant was added to treat them to replicate the unfavorable healing environment. The angiogenesis experiments showed that SPARC could promote HUVEC angiogenesis more effectively, as evidenced by more vascular nodes and an increase in vessel lengths (Figure 2E–G). In addition, CCK-8 results revealed that SPARC significantly promoted HUVEC proliferation (Figure 2H), while scratch experiments likewise showed that SPARC significantly enhanced HUVEC migration (Figure 2J and Figure S1b). Meanwhile, SPARC also promoted the proliferation and migration of HaCaT (Figure 2I,K and Figure S1c).

### 3.2. SPARC-ADSCs Proteomic Sequencing

**Figure 3:**
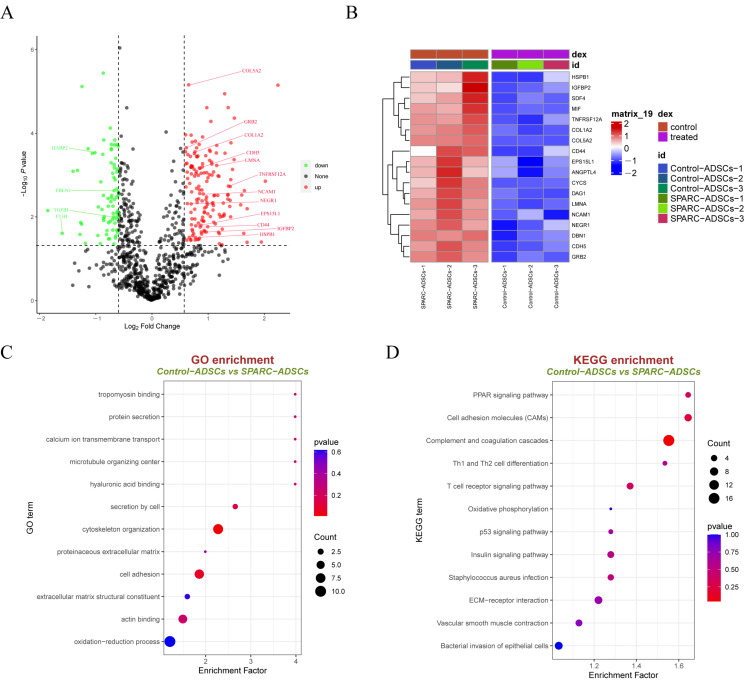
Quantitative proteomics sequencing analysis of ADSCs reveals differentially expressed proteins and pathways affected by SPARC. (**A**) Volcano plot of differentially expressed proteins identified by quantitative proteomics sequencing of ADSCs stably expressing SPARC; (**B**) Heatmap of quantitative proteomics sequencing data showing representative differentially expressed proteins regulated by SPARC; (**C**) SPARC-GO enrichment analysis bubble map of differentially expressed proteins of ADSCs and Control-ADSCs; (**D**) KEGG enrichment analysis bubble map of differentially expressed proteins of SPARC-ADSCs and Control-ADSCs.

To explore the target proteins and downstream signaling pathways regulated by SPARC, we analyzed DIA quantitative proteomics sequencing data on oe-SPARC-ADSC and Control-ADSC cell supernatants. The sequencing results showed that 167 proteins were significantly up-regulated and 74 proteins were significantly down-regulated in the oe-SPARC-ADSC group (Figure 3A). The heatmap showed representative genes positively regulated by SPARC. Including COL1A2, HSPB1, NCAM1, ANGPTL4, and others as shown in Figure 3B. GO analysis of up- and down-regulated differentially expressed proteins revealed that the pathways enriched mainly include cell adhesion, hyaluronan binding, microtubule organization center, and protein extracellular matrix (Figure 3C). KEGG analysis of these up- and down-regulated differentially expressed proteins enriched pathways mainly including complement and coagulation cascades, Th1 and Th2 cell differentiation, ECM-receptor interaction, and vascular smooth muscle contraction (Figure 3D).

### 3.3. SPARC-ADSCs Promote Healing of Skin Wound

**Figure 4:**
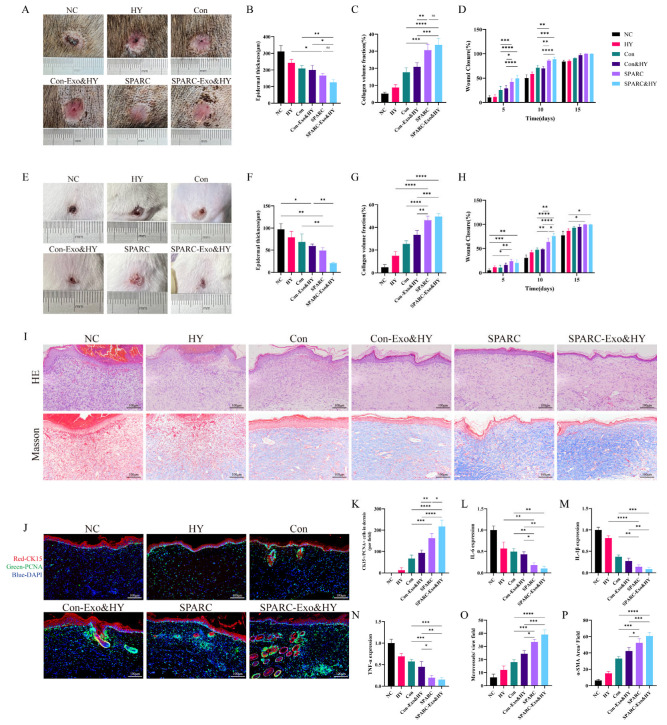
SPARC-ADSCs promote the healing of skin wounds. (**A**) Healing of dog wounds on day 15; (**B**) Quantitative analysis of dog epidermal thickness; (**C**) Quantitative analysis of dog collagen volume fraction; (**D**) Quantification of dog wound-healing rate; (**E**) Healing of mouse wounds on day 15; (**F**) Quantitative analysis of mouse epidermal thickness; (**G**) Quantitative analysis of mouse collagen volume fraction; (**H**) Quantification of mouse wound-healing rate; (**I**) Hematoxylin and eosin (H&E) staining and Masson staining of paraffin sections of mouse skin tissues; (**J**) Double immunofluorescence staining of PCNA (green) and CK15 (red) in paraffin sections of mouse skin tissues showing the proliferation status of hair follicle stem cells; (**K**) Quantitative analysis of immunofluorescence staining results; (**L**–**P**) Quantitative analysis of IL-6, IL-1β, TNF-α, CD31, α-SMA positive expression levels. ns: Difference is not significant; *: *p* < 0.05; **: *p* < 0.01; ***: *p* < 0.001; ****: *p* < 0.0001.

Subsequently, the dog wound model was used to confirm the pro-healing impact of oe-SPARC-ADSCs. A circular model of a skin wound approximately 1.5 cm in diameter was made on the dorsal side of the dogs. Every three days, oe-SPARC-ADSCs were subcutaneously injected around the lesion. The skin wound area of the SPARC and SPARC-Exo&HY treatment groups was less than that of the remaining control group at every time point of the wound (Figure S2a), and on the 15th day, the wounds of the SPARC and SPARC-Exo&HY groups were completely healed, as shown in the figure (Figure 4A). The degree of wound re-epithelialization was evaluated by HE staining, and at day 15 post-wound, the SPARC vs. SPARC-Exo&HY groups’ wounds had a thinner epidermal thickness and a more complete degree of re-epithelialization than the wounds in the other control groups, as shown in Figure 4B and Figure S2c. Collagen arrangement and distribution are important factors for the determination of the quality of wound healing. Masson staining showed the collagen in the wounds of the different treatment groups, and the quantitative results showed that the collagen content in the SPARC vs. SPARC-Exo&HY groups was significantly higher than that in the control group (Figure 4C).

The mouse wound model was selected to verify the oe-SPARC-ADSC pro-healing effect. The mice’s backs were used to create a circular skin whole-layer wound model with a diameter of 1 cm. The gross images of each group’s wounds at day 15 are shown in Figure 4E, which demonstrates that the SPARC and SPARC-Exo&HY groups’ skin wound area was substantially smaller than that of the remaining control group and that their wound-healing rate was nearly 100% (Figure 4H). We further performed H&E and Masson staining on the wound samples to assess the degree of epithelialization, granulation formation, and collagen deposition in the wound bed at the histological level on day 15 post-wound. The H&E staining results showed that the granulation tissues in the SPARC vs. SPARC-Exo&HY groups exhibited good healing and complete re-epithelialization (Figure 4I). The collagen fibers in SPARC and SPARC-Exo&HY groups were regular and well organized, as seen in the Masson results. Both H&E and Masson staining showed that the therapeutic effect of SPARC and SPARC-Exo&HY groups was satisfactory and suitable for the treatment of common wounds.

Cytokeratin 15 (CK15) can be used as a marker for hair follicle regeneration due to its high expression in hair follicle stem cells. We performed double immunofluorescence staining of skin tissues for CK15 and Proliferating Cell Nuclear Antigen (PCNA) (Figure 4J and Figure S2e), and the results showed that SPARC and SPARC-Exo&HY groups significantly increased the number of CK15+/PCNA+ cells in the dermis. Thus, we concluded that SPARC promotes hair follicle regeneration during wound healing.

Inflammation is a complicated and important factor in wound healing. To effectively manage wounds, the inflammatory response must be balanced such that it effectively eliminates infection while remaining moderate enough to encourage rapid and efficient wound healing. IL-6, IL-1β, and TNF-α were used to characterize the inflammatory response in wounds (Figure S2d). Immunohistochemical staining was used to assess each group’s expression levels of IL-6, IL-1β, and TNF-α 15 days after the wound (Figure 4L–N), and subsequent quantitative analysis revealed that both SPARC and SPARC-Exo&HY groups significantly reduced the expression of wound inflammatory factors.

Blood vessels provide nutrients and oxygen for the whole process of wound healing and are crucial in the process of wound repair. CD31 represents neovascularization in the wound, and α-smooth muscle actin (α-SMA) represents mature blood vessels. On day 15 day post wound, CD31 and α-SMA immunohistochemical staining were performed separately. The results showed that the vascular content of both SPARC and SPARC-Exo&HY groups was superior to that of the remaining control group (Figure 4O,P).

According to our results, animals’ skin wound healing was considerably accelerated by either subcutaneous injection of ADSCs overexpressing SPARC alone or by the use of hydrogel loaded with SPARC exosomes. The therapeutic effect of hydrogel loaded with SPARC exosomes was higher than that of the cell-only injection.

### 3.4. SPARC-ADSCs Promote Healing of Diabetic Skin Wound

**Figure 5:**
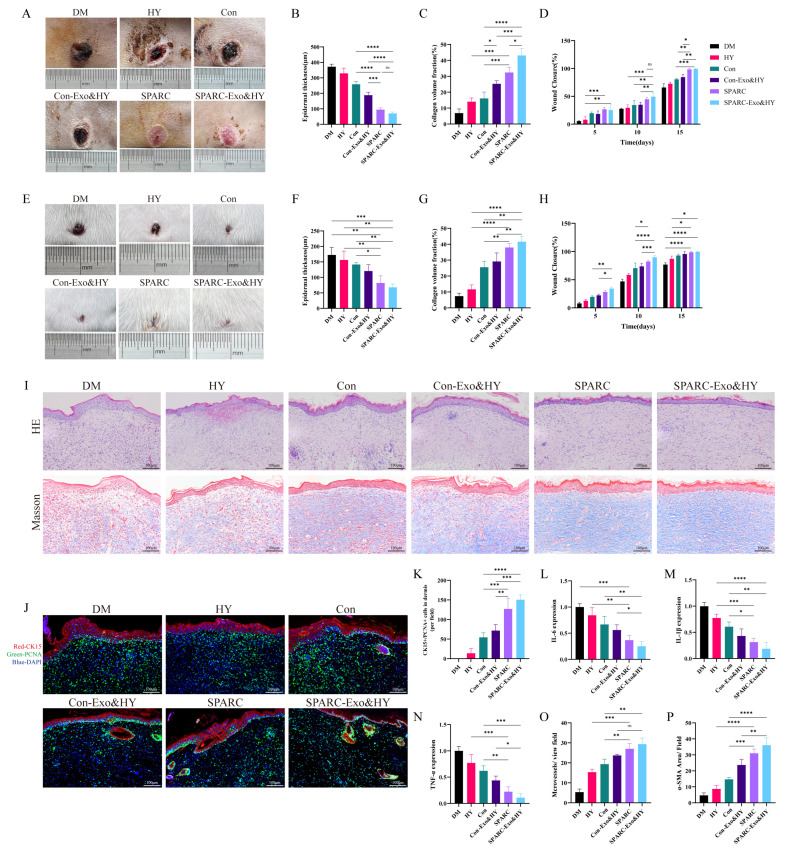
SPARC-ADSCs promote the healing of diabetic skin wounds. (**A**) Healing of diabetic dog wounds on day 15; (**B**) Quantitative analysis of HE-stained epidermal thickness of diabetic dog skin tissue sections; (**C**) Quantitative analysis of Masson-stained collagen volume fraction of diabetic dog skin tissue sections; (**D**) Quantification of healing rate of diabetic dog wounds; (**E**) Healing of diabetic mice wounds on day 15; (**F**) Quantification of epidermal thickness by HE staining of diabetic mouse skin tissue sections; (**G**) Quantification of collagen volume fraction by HE staining of diabetic mouse skin tissue sections; (**H**) Quantification of wound-healing rate in diabetic mice; (**I**) Hematoxylin and eosin (H&E) staining and Masson staining of paraffin sections of diabetic mouse skin tissues; (**J**) Paraffin sections of diabetic mouse skin tissues PCNA (green) and CK15 (red) double immunofluorescence staining showing the proliferative status of hair follicle stem cells; (**K**) Quantification of immunofluorescence staining results; (**L**–**P**) Quantification of IL-6, IL-1β, TNF-α, CD31, and α-SMA positive expression levels. ns: Difference is not significant; *: *p* < 0.05; **: *p* < 0.01; ***: *p* < 0.001; ****: *p* < 0.0001.

Diabetic animals have difficulty in wound healing due to high blood glucose levels and circulatory problems, resulting in chronic wounds and increased risk of infection. Diabetic dogs wound model used to validate the pro-healing effect of oe-SPARC-ADSCs. The diabetic model was established. A single high dose of STZ administered to dogs showed positive oral glucose tolerance test results (Figure S3a,b), and blood glucose values greater than 6.7 mmol/L were measured for 7 consecutive days. Subsequently, a 1.5 cm circular model of full skin damage was made on the dog’s back, and every three days, 2 million cells or 45 μg of exosomes were injected subcutaneously surrounding the wound. On day 15, wound tissue samples were harvested from the neo-formed skin of DM dogs. On day 15, the SPARC and SPARC-Exo&HY treated wounds healed completely, as shown (Figure 5A), and the healing rate of the SPARC and SPARC-Exo&HY groups was significantly higher than the rest of the controls (Figure 5D). H&E and Masson staining were used on diabetic dog wound samples (Figure S3g), and the statistical results showed that the SPARC vs. SPARC-Exo&HY groups were significantly recovered compared to the control group, both in terms of the degree of re-epithelialization of the epidermis and collagen deposition (Figure 5B,C). Subsequently, Diabetic mouse wound models were used to observe pro-healing effects. The blood glucose values measured from the tail vein of the mice exceeded 11.1 mmol/L, and the results of the oral glucose tolerance test (OGTT) were positive (Figure S3c,d). On day 15 post-wound, from the gross wound images, it was found that the wounds in the SPARC and SPARC-Exo&HY groups completely healed, and the quantitative results showed that SPARC was effective in promoting skin wound healing in diabetic mice (Figure 5E,H). H&E and Masson staining were used for histological observation to assess the degree of epithelialization, granulation tissue formation, and collagen deposition on day 15. The results of H&E staining showed that the granulation tissues in the SPARC and the SPARC-Exo&HY group exhibited good healing and were adequately epithelialized (Figure 5I). From the results of Masson staining, the collagen fibers in the SPARC group and SPARC-Exo&HY group were well arranged.

Double immunofluorescence staining of CK15 with PCNA was performed on skin tissue sections from diabetic mice (Figure 5J and Figure S3i), which showed that the number of CK15+/PCNA+ cells in the dermis was significantly increased in the SPARC and the SPARC-Exo&HY group. It is concluded that SPARC could be more effective in promoting hair follicle regeneration during the wound-healing process.

The inflammatory factors post-wound in diabetic animals were significantly higher than those in normal healthy animals. Thus, continued to select IL-6, IL-1β, and TNF-α to characterize the wound inflammatory response (Figure S3h). At 15 days post-wound, the expression levels of IL-6, IL-1β, and TNF-α in each group, which were examined by immunohistochemical staining (Figure 5L–N), and quantitative analysis revealed that the SPARC and the SPARC-Exo&HY group showed significant reductions in traumatic inflammatory factor expression.

Immunohistochemical staining for CD31 and α-SMA was performed, and the results showed that the vascular content of both the SPARC and the SPARC-Exo&HY group was significantly better than that of the control groups (Figure 5O,P).

Our findings showed that either ADSCs overexpressing SPARC alone by subcutaneous injection or the use of hydrogels containing SPARC exosomes effectively promotes wound healing in animals. In addition, the application of hydrogels loaded with SPARC exosomes was more effective than the cell-only injection.

### 3.5. SPARC-ADSCs Promote Healing of Aging Skin Wound

**Figure 6:**
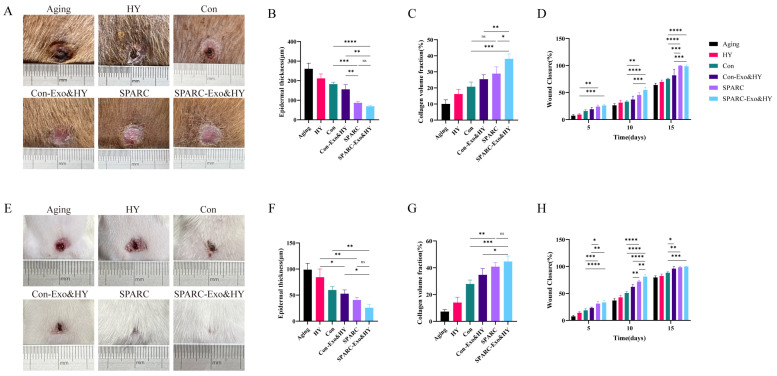

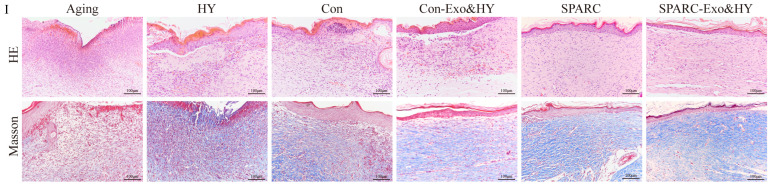
SPARC-ADSCs promote the healing of senescent skin wounds. (**A**) Healing of senescent dog wounds on day 15; (**B**) Quantification of HE-stained epidermal thickness of senescent dog skin tissue sections; (**C**) Quantification of Masson-stained collagen volume fraction of senescent dog skin tissue sections; (**D**) Quantification of healing rate of senescent dog wounds; (**E**) Healing of senescent mouse wounds on day 15; (**F**) Senescent mouse Quantification of epidermal thickness by HE staining of skin tissue sections; (**G**) Quantification of collagen volume fraction by Masson staining of skin tissue sections from aging mice; (**H**) Quantification of wound-healing rate in aging mice; (**I**) Hematoxylin and eosin (H&E) staining and Masson staining of paraffin sections of skin tissue from aging mice. ns: Difference is not significant; *: *p* < 0.05; **: *p* < 0.01; ***: *p* < 0.001; ****: *p* < 0.0001.

Wound healing is impaired or delayed as individuals age. There is evidence that the effects of age on the epidermis and dermis, mainly changes in the dermis, lead to changes in skin integrity and increased epidermal susceptibility to wound [30]. Therefore, prompt management therapy is required for aging animals with skin wounds. Furthermore, aging dog models were used to evaluate the pro-healing effects of oe-SPARC-ADSCs. Thus, six circular skin excisions of the same size were performed on the dorsal side of aging dogs, and skin healing was recorded as shown in Figure 6A. The wound-healing rate increased with time in all groups. The aging group showed the slowest healing rate, while the SPARC and SPARC-Exo&HY groups showed higher healed wounds at day 15, as shown in the figure (Figure 6D). HE staining of skin samples (Figure S4c) showed the thinnest epidermal and complete re-epithelialization in the SPARC compared to SPARC-Exo&HY groups at day 15, as shown in Figure 5B. After 15 days, Masson staining of the repaired skin samples revealed nearly no blue spots in the aging group, indicating almost no collagen deposition. Both the SPARC and SPARC-Exo&HY groups showed higher collagen deposition and collagen fiber repair as compared to the control group. The pro-healing effect of oe-SPARC-ADSCs was validated by a senescent mouse model.

At 15 days after the wound, the wounds in the SPARC and SPARC-Exo&HY groups were completely healed, and the coat had covered them (Figure 6E). The healing rate of the wounds increased with time, and the healing rate of the SPARC and SPARC-Exo&HY groups was significantly higher compared to the control groups (Figure 6H). By HE staining of skin tissue sections of senescent mice, the degree of re-epithelialization was improved in both SPARC and SPARC-Exo&HY groups compared to the control group (Figure 6I,F). Subsequent Masson staining showed significant collagen deposition in the SPARC and SPARC-Exo&HY groups, as shown in the figure (Figure 6I,G). Our results indicated that SPARC improved the pro-healing effect on skin wounds in aging dogs.

## 4. Discussion

One of the reasons wounds need to heal effectively is to reestablish the body’s barrier function, which prevents more serious damage and infection. Wound healing is a complex biological process that generally involves four phases: coagulation and hemostasis, inflammation, cell proliferation, and tissue remodeling [6]. The inflammatory phase involves platelet activation, which results in the formation of a fibrin clot and secretion of chemokines, followed by the recruitment of neutrophils (to clear foreign particles, including bacteria, from the wound), T-cells (to maintain inflammation and to recruit monocytes), and the activation of macrophages (to clear debris) [31,32]. M2 macrophages are involved in the synthesis of anti-inflammatory mediators and the production of extracellular matrix (ECM), in the adult initiation of fibroblast proliferation, and the process of angiogenesis [33,34]. The role of the inflammatory phase is to remove foreign cells and molecules from the wound before rebuilding the skin. In rodents, wounds heal mainly by epithelial contraction, whereas in humans, re-epithelialization accounts for 80% of wound closure [35]. Re-epithelialization begins several hours after wounding through the transformation of cobblestone-like quiescent keratinocytes into flattened migratory keratinocytes [36].

The surface of EV derived from MSCs contains tissue factor and phosphatidylserine, which can trigger coagulation. Expression of these two factors triggers a thrombotic response that increases clot formation [37]. It has been shown that transplantation of MSCs into the wound during the early stages of wound healing promotes a faster coagulation response and thus faster progression to the next stage of wound healing. MSCs secrete a variety of growth factors and cytokines that modulate the response of neutrophils, macrophages, and lymphocytes [38]. It has been shown that MSCs modulate macrophage responses. Zhang et al. demonstrated that MSCs were able to polarize macrophages from pro-inflammatory M1 to a reparative/anti-inflammatory M2-M2-activated state during skin wound healing [39]. Switching macrophage activation from an M1 inflammatory phenotype to an M2 restorative/anti-inflammatory phenotype is a critical step in wound healing and controlling inflammation. Final wound contraction and completion of epithelialization require the involvement of fibroblasts and the proliferation and recovery of epithelial cells. Treatment with MSCs enhances fibroblast survival and migration, increases fibroblast ECM deposition, and enhances healing [40]. MSC-derived exosomes also lead to collagen deposition, exert an antifibrotic effect in proliferative scarring, and promote fibroblast proliferation and migration [41,42]. However, there are still many problems with the use of MSCs for wound treatment, including whether the animal itself suffers from an underlying disease, the surrounding environment for wound healing, and various unfavorable factors in vivo and in vitro that affect the therapeutic effect of MSCs and reduce the activity of MSCs. Moreover, 80–90% of MSCs die within 72 h after transplantation [43]. Therefore, it is important to improve the survival rate of MSCs after transplantation and their ability to migrate directionally in the trauma.

SPARC promotes collagen deposition in tissues and plays an important role in extracellular matrix regulation [15]. Collagen fiber scaffolds provide structural integrity to the extracellular matrix of connective tissues and basement membranes, and SPARC expression is required for collagen fiber deposition in the basal lamina [44]. In addition, SPARC promotes angiogenesis by regulating vascular endothelial growth factor (VEGF), fibroblast growth factor 2 (FGF2), and others [45]. In this study, we used SPARC to modify MSCs. The results showed that SPARC has antioxidant effects, which can attenuate the damage of oxidative stress on MSCs, as well as promote the proliferation and migration ability of vascular endothelial cells and keratinocytes in an oxidative stress environment, which helps to maintain the healthy state of peri-wound tissues and promote healing. Analysis of proteomic sequencing data further confirmed that SPARC promotes cell adhesion and hyaluronan binding, Th1 and Th2 cell differentiation, and other important biological processes in ADSCs. This confirms the idea that SPARC enhances the repair capacity of ADSCs in wound healing.

We validated the therapeutic effect of oe-SPARC-ADSCs on wound healing using a total of three model animals: normal, diabetic, and senescent. Our results showed that SPARC-modified ADSCs were able to promote collagen deposition and angiogenesis in wounds, accelerate the degree of re-epithelialization, and reduce inflammatory indicators, as well as hair follicle neogenesis. This suggests that SPARC-modified ADSCs can accelerate wound healing in skin-injured dogs. Throughout the treatment period and subsequent observation, no animals in any experimental group exhibited clinical complications directly attributable to the injection of cells or exosomes, such as severe inflammation or necrosis at the injection site, signs of systemic infection, or behavioral abnormalities. Body weight, food intake, and basic activities remained normal in all animals. These results indicate that the local application of ADSCs and their derivatives demonstrated favorable safety and tolerability under the experimental conditions.

In our study, SPARC-modified ADSCs showed efficiency in dog wound healing. However, there are some limitations to consider in this study. The sample size was relatively small, and the study period was brief. Therefore, the generalizability of the study results urgently needs more large-scale and long-term clinical justification. In addition, other aspects, such as the mechanism of the effect of SPARC modification on ADSCs, still need to be studied and explored in depth for a more comprehensive understanding of its mechanism of action.

This preclinical study demonstrates that SPARC overexpression enhances the therapeutic potential of ADSCs and their exosomes in skin wound repair. Using three distinct animal models (normal, diabetic, and aged dogs), we found that SPARC-modified ADSCs significantly improved key healing parameters—including re-epithelialization, collagen deposition, angiogenesis, and modulation of peri-wound inflammation—compared to control ADSCs or hydrogel alone. In vitro analyses further revealed that SPARC augmentation boosted cellular resistance to oxidative stress and enhanced the proliferative and migratory capacities of HUVEC and HaCaT under stress conditions. Proteomic profiling indicated that SPARC’s mechanistic role may involve the regulation of pathways related to cell adhesion, hyaluronan binding, complement/coagulation cascades, and vascular regeneration.

While these results highlight the promising role of SPARC in optimizing ADSC-based therapies, several important limitations must be acknowledged. First, the study was conducted in controlled animal models, which may not fully replicate the complexity of human chronic wounds. Second, the long-term safety, optimal dosing, and potential immunogenicity of SPARC-modified ADSCs remain to be systematically evaluated. Third, the precise molecular mechanisms through which SPARC exerts its observed effects warrant further elucidation.

Therefore, future studies should focus on:

(1) Validating these findings in larger animal models or human-relevant ex vivo systems;
(2) Conducting long-term safety and efficacy studies, including assessments of potential tumorigenicity or off-target effects;
(3) Elucidating the downstream signaling networks and extracellular vesicle cargo alterations driven by SPARC overexpression; and
(4) Integrating recent advances in biomaterial-based delivery systems [46] to enhance localized retention and controlled release.

In summary, this work provides a foundational preclinical evidence base for SPARC-enhanced ADSC therapy. Translating these findings into clinical applications will require rigorous validation through further mechanistic research and translational development.

## Data Availability

The original contributions presented in this study are included in the article/Supplementary Materials. Further inquiries can be directed to the corresponding author.

## Supplementary Materials

The following supporting information can be downloaded at: https://www.mdpi.com/article/10.3390/vetsci13030222/s1, Figure S1: SPARC enhances the resistance of MSCs, endothelial, and keratinocytes to injury. (a) oe-SPARC-ADSC bright field photo; (b) HUVEC scratch experiment; (c) HaCaT scratch experiment; Figure S2: SPARC-ADSCs promote healing of skin wound. (a) Gross image of canine wound healing; (b) Gross image of mouse wound healing; (c) Hematoxylin and eosin (H&E) staining and Masson staining of paraffin sections of canine skin tissues; (d) Immunohistochemical staining of paraffin sections of mouse skin tissues for IL-6, IL-1β, TNF-α, CD31, α-SMA; (e) Double immunofluorescence staining of PCNA and CK15 in paraffin sections of mouse skin tissue showing the proliferative status of hair follicle stem cells. Figure S3: SPARC-ADSCs promote healing of diabetic skin wounds. (a) Diabetic dog blood glucose values; (b) Diabetic dog oral glucose tolerance test (OGTT); (c) Diabetic mouse blood glucose values; (d) Diabetic mouse oral glucose tolerance test (OGTT); (e) Diabetic dog trauma healing macroscopic image; (f) Diabetic mouse trauma healing macroscopic image; (g) Diabetic dog skin tissueparaffin sections stained with hematoxylin and eosin (H&E) and stained with Masson stain; (h) Immunohistochemical staining of paraffin sections of skin tissues of diabetic mice with IL-6, IL-1β, TNF-α, CD31, α-SMA; (i) Double immunofluorescence staining of paraffin sections of skin tissues of diabetic mice with PCNA and CK15 showing the proliferative state of hair follicle stem cells. Figure S4: SPARC-ADSCs promote healing of senescent skin injuries. (a) Gross image of senescent canine trauma healing; (b) Gross image of senescent mouse trauma healing; (c) Hematoxylin and eosin (H&E) staining and Masson staining of senescent canine skin tissues in paraffin sections.

## References

[1] Zhong S.P., Zhang Y.Z., Lim C.T. (2010). Tissue scaffolds for skin wound healing and dermal reconstruction. Wiley Interdiscip. Rev. Nanomed. Nanobiotechnol..

[2] Blair M.J., Jones J.D., Woessner A.E., Quinn K.P. (2020). Skin Structure-Function Relationships and the Wound Healing Response to Intrinsic Aging. Adv. Wound Care.

[3] Wong R., Geyer S., Weninger W., Guimberteau J.C., Wong J.K. (2016). The dynamic anatomy and patterning of skin. Exp. Dermatol..

[4] Bouwstra J.A., Honeywell-Nguyen P.L. (2002). Skin structure and mode of action of vesicles. Adv. Drug Deliv. Rev..

[5] Murphree R.W. (2017). Impairments in Skin Integrity. Nurs. Clin. N. Am..

[6] Velnar T., Bailey T., Smrkolj V. (2009). The wound healing process: An overview of the cellular and molecular mechanisms. J. Int. Med. Res..

[7] Han G., Ceilley R. (2017). Chronic Wound Healing: A Review of Current Management and Treatments. Adv. Ther..

[8] Tam J., Wang Y., Farinelli W.A., Jiménez-Lozano J., Franco W., Sakamoto F.H., Cheung E.J., Purschke M., Doukas A.G., Anderson R.R. (2013). Fractional Skin Harvesting: Autologous Skin Grafting without Donor-site Morbidity. Plast. Reconstr. Surg. Glob. Open.

[9] Heyboer M., Sharma D., Santiago W., McCulloch N. (2017). Hyperbaric Oxygen Therapy: Side Effects Defined and Quantified. Adv. Wound Care.

[10] Ding D.C., Shyu W.C., Lin S.Z. (2011). Mesenchymal stem cells. Cell Transplant..

[11] Uccelli A., Moretta L., Pistoia V. (2008). Mesenchymal stem cells in health and disease. Nat. Rev. Immunol..

[12] Kou Z., Li B., Aierken A., Tan N., Li C., Han M., Jing Y., Li N., Zhang S., Peng S. (2023). Mesenchymal Stem Cells Pretreated with Collagen Promote Skin Wound-Healing. Int. J. Mol. Sci..

[13] Rosset E.M., Bradshaw A.D. (2016). SPARC/osteonectin in mineralized tissue. Matrix Biol..

[14] Ghanemi A., Yoshioka M., St-Amand J. (2021). Secreted Protein Acidic and Rich in Cysteine as A Regeneration Factor: Beyond the Tissue Repair. Life.

[15] Bradshaw A.D. (2009). The role of SPARC in extracellular matrix assembly. J. Cell Commun. Signal..

[16] Rentz T.J., Poobalarahi F., Bornstein P., Sage E.H., Bradshaw A.D. (2007). SPARC regulates processing of procollagen I and collagen fibrillogenesis in dermal fibroblasts. J. Biol. Chem..

[17] Murphy-Ullrich J.E., Sage E.H. (2014). Revisiting the matricellular concept. Matrix Biol..

[18] MacArthur Clark J.A., Sun D. (2020). Guidelines for the ethical review of laboratory animal welfare People’s Republic of China National Standard GB/T 35892-2018 [Issued 6 February 2018 Effective from 1 September 2018]. Anim. Model. Exp. Med..

[19] Li C., Li B., Han M., Tian H., Gao J., Han D., Ling Z., Jing Y., Li N., Hua J. (2024). SPARC overexpression in allogeneic adipose-derived mesenchymal stem cells in dog dry eye model induced by benzalkonium chloride. Stem Cell Res. Ther..

[20] Jin X., Dai Y., Xin L., Ye Z., Chen J., He Q., Chen X., Xu X., Song G., Yu X. (2023). ADSC-derived exosomes-coupled decellularized matrix for endometrial regeneration and fertility restoration. Mater. Today Bio.

[21] Zhao X., Li X., Wang Y., Guo Y., Huang Y., Lv D., Lei M., Yu S., Luo G., Zhan R. (2023). Stability and biosafety of human epidermal stem cell for wound repair: Preclinical evaluation. Stem Cell Res. Ther..

[22] He W., Qin D., Li B., Zhang H., Cheng X., Sun J., Hua J., Peng S. (2021). Immortalized canine adipose-derived mesenchymal stem cells alleviate gentamicin-induced acute kidney injury by inhibiting endoplasmic reticulum stress in mice and dogs. Res. Vet. Sci..

[23] Aierken A., Li B., Liu P., Cheng X., Kou Z., Tan N., Zhang M., Yu S., Shen Q., Du X. (2022). Melatonin treatment improves human umbilical cord mesenchymal stem cell therapy in a mouse model of type II diabetes mellitus via the PI3K/AKT signaling pathway. Stem Cell Res. Ther..

[24] Zaqout S., Becker L.L., Kaindl A.M. (2020). Immunofluorescence Staining of Paraffin Sections Step by Step. Front. Neuroanat..

[25] Gong L., Lei Y., Liu Y., Tan F., Li S., Wang X., Xu M., Cai W., Du B., Xu F. (2019). Vaccarin prevents ox-LDL-induced HUVEC EndMT, inflammation and apoptosis by suppressing ROS/p38 MAPK signaling. Am. J. Transl. Res..

[26] Zhang M.F., Wan S.C., Chen W.B., Yang D.H., Liu W.Q., Li B.L., Aierken A., Du X.M., Li Y.X., Wu W.P. (2023). Transcription factor Dmrt1 triggers the SPRY1-NF-κB pathway to maintain testicular immune homeostasis and male fertility. Zool Res..

[27] Jiang B.W., Zhang W.J., Wang Y., Tan L.P., Bao Y.L., Song Z.B., Yu C.L., Wang S.Y., Liu L., Li Y.X. (2020). Convallatoxin induces HaCaT cell necroptosis and ameliorates skin lesions in psoriasis-like mouse models. Biomed. Pharmacother..

[28] Yang D., Wei Y., Lu Q., Qin D., Zhang M., Du X., Xu W., Yu X., He C., Li N. (2021). Melatonin alleviates LPS-induced endoplasmic reticulum stress and inflammation in spermatogonial stem cells. J. Cell. Physiol..

[29] Vernon R.B., Gooden M.D., Chan C.K., Workman G., Obika M., Wight T.N. (2021). Autocrine Hyaluronan Influences Sprouting and Lumen Formation During HUVEC Tubulogenesis In Vitro. J. Histochem. Cytochem..

[30] Bonifant H., Holloway S. (2019). A review of the effects of ageing on skin integrity and wound healing. Br. J. Community Nurs..

[31] Havran W.L., Jameson J.M. (2010). Epidermal T cells and wound healing. J. Immunol..

[32] Su Y., Richmond A. (2015). Chemokine Regulation of Neutrophil Infiltration of Skin Wounds. Adv. Wound Care.

[33] Sindrilaru A., Scharffetter-Kochanek K. (2013). Disclosure of the Culprits: Macrophages-Versatile Regulators of Wound Healing. Adv. Wound Care.

[34] Brancato S.K., Albina J.E. (2011). Wound macrophages as key regulators of repair: Origin, phenotype, and function. Am. J. Pathol..

[35] Volk S.W., Bohling M.W. (2013). Comparative wound healing—Are the small animal veterinarian’s clinical patients an improved translational model for human wound healing research?. Wound Repair Regen..

[36] Rittié L. (2016). Cellular mechanisms of skin repair in humans and other mammals. J. Cell Commun. Signal..

[37] Chance T.C., Rathbone C.R., Kamucheka R.M., Peltier G.C., Cap A.P., Bynum J.A. (2019). The effects of cell type and culture condition on the procoagulant activity of human mesenchymal stromal cell-derived extracellular vesicles. J. Trauma Acute Care Surg..

[38] Ohtaki H., Ylostalo J.H., Foraker J.E., Robinson A.P., Reger R.L., Shioda S., Prockop D.J. (2008). Stem/progenitor cells from bone marrow decrease neuronal death in global ischemia by modulation of inflammatory/immune responses. Proc. Natl. Acad. Sci. USA.

[39] Zhang Q.Z., Su W.R., Shi S.H., Wilder-Smith P., Xiang A.P., Wong A., Nguyen A.L., Kwon C.W., Le A.D. (2010). Human gingiva-derived mesenchymal stem cells elicit polarization of m2 macrophages and enhance cutaneous wound healing. Stem Cells.

[40] Yates C.C., Rodrigues M., Nuschke A., Johnson Z.I., Whaley D., Stolz D., Newsome J., Wells A. (2017). Multipotent stromal cells/mesenchymal stem cells and fibroblasts combine to minimize skin hypertrophic scarring. Stem Cell Res. Ther..

[41] Li Y., Zhang J., Shi J., Liu K., Wang X., Jia Y., He T., Shen K., Wang Y., Liu J. (2021). Exosomes derived from human adipose mesenchymal stem cells attenuate hypertrophic scar fibrosis by miR-192-5p/IL-17RA/Smad axis. Stem Cell Res. Ther..

[42] Zhang W., Bai X., Zhao B., Li Y., Zhang Y., Li Z., Wang X., Luo L., Han F., Zhang J. (2018). Cell-free therapy based on adipose tissue stem cell-derived exosomes promotes wound healing via the PI3K/Akt signaling pathway. Exp. Cell Res..

[43] Liu X.B., Wang J.A., Ogle M.E., Wei L. (2009). Prolyl hydroxylase inhibitor dimethyloxalylglycine enhances mesenchymal stem cell survival. J. Cell. Biochem..

[44] Martinek N., Shahab J., Sodek J., Ringuette M. (2007). Is SPARC an evolutionarily conserved collagen chaperone?. J. Dent. Res..

[45] Rivera L.B., Bradshaw A.D., Brekken R.A. (2011). The regulatory function of SPARC in vascular biology. Cell. Mol. Life Sci..

[46] Yu H., Zhang J., Yang L., Tian Y., Milne C., Jin P., Li Q., Song R., Wang W. (2025). MSC-derived exosomes injectable hyaluronic acid hydrogel for enhanced chronic wound healing. J. Control. Release.

