# Supplementary material for: Clinical Efficacy of SPARC-Modified Mesenchymal Stem Cells for the Treatment of Dog Skin Wounds
Source: Vet Sci. 2026 Feb 26;13(3):222. doi: 10.3390/vetsci13030222 (PMC13030800; doi:10.3390/vetsci13030222)
Supplement: Supplementary file 1 [file vetsci-13-00222-s001.zip › vetsci-4118021-supplementary.pdf]

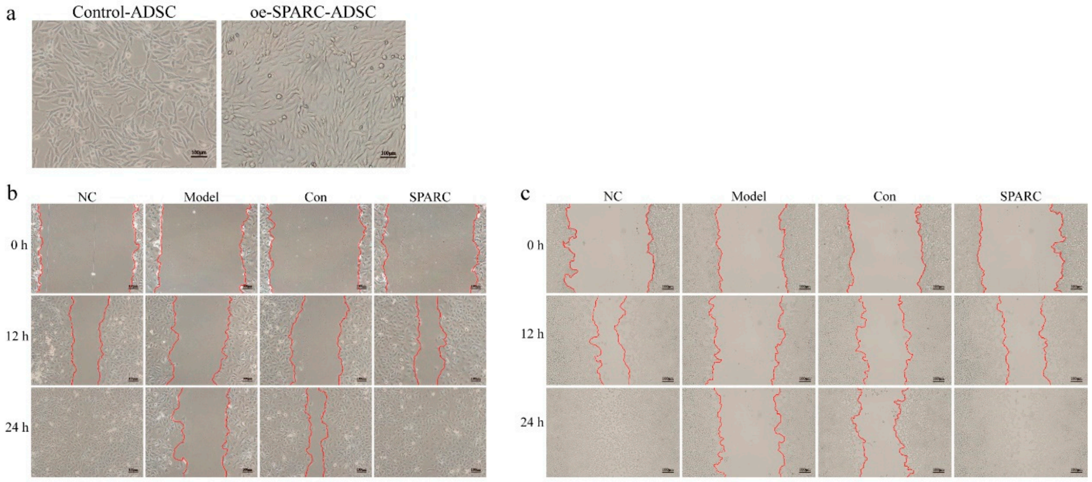

Fig. S1 SPARC enhances the resistance of MSCs, endothelial and keratinocytes to injury. (a) oe-SPARC-ADSC bright field photo; (b) HUVEC scratch experiment; (c) HaCaT scratch experiment

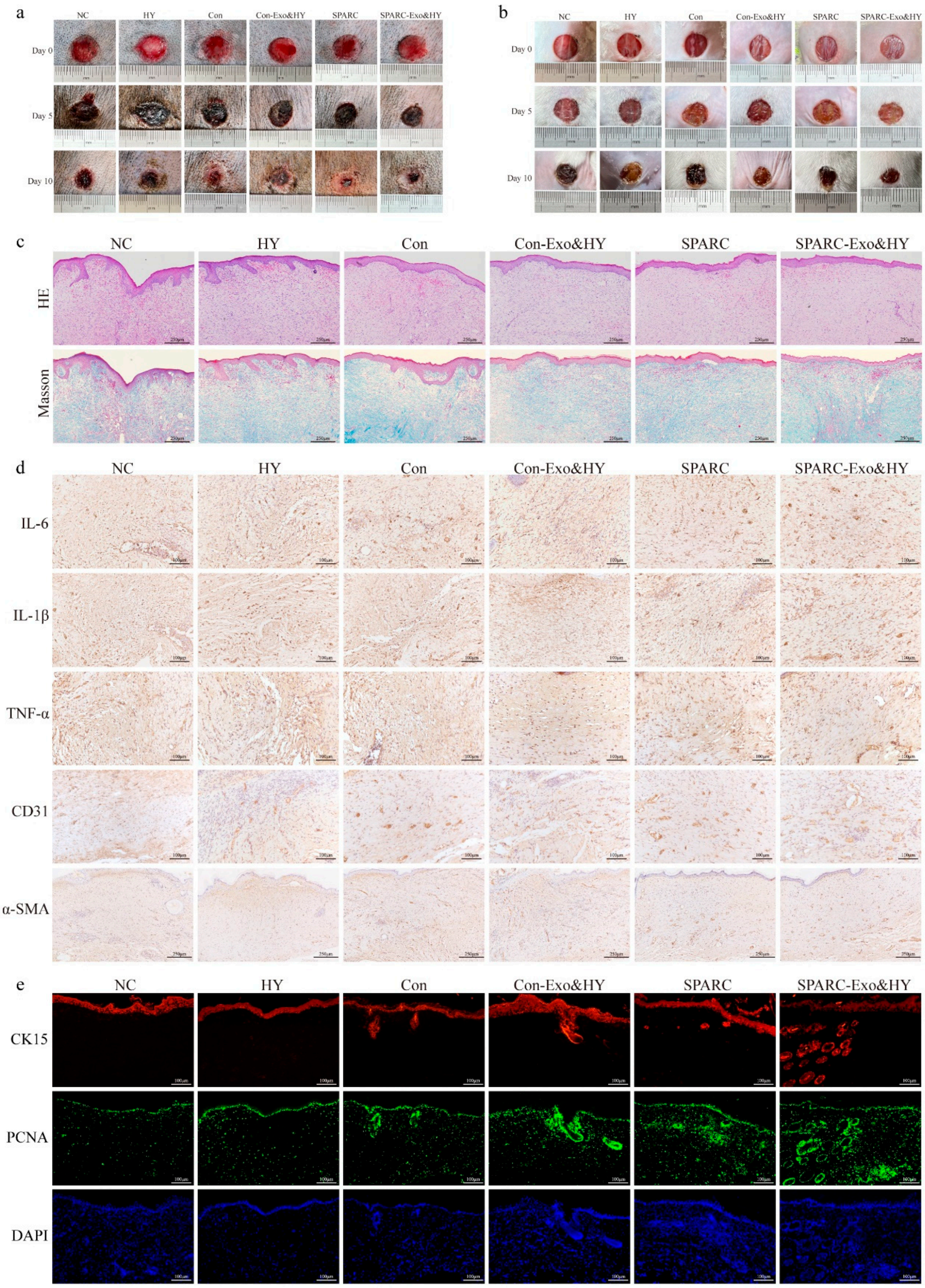

Fig. S2 SPARC-ADSCs promote healing of skin wound. (a) Gross image of canine wound healing; (b) Gross image of mouse wound healing; (c) Hematoxylin and eosin (H&E) staining and Masson staining of paraffin sections of canine skin tissues; (d) Immunohistochemical staining of paraffin sections of mouse skin tissues for IL-6, IL-1β, TNF-α, CD31, α-SMA;(e) Double immunofluorescence staining of PCNA and CK15 in paraffin sections of mouse skin tissue showing the proliferative status of hair follicle stem cells.

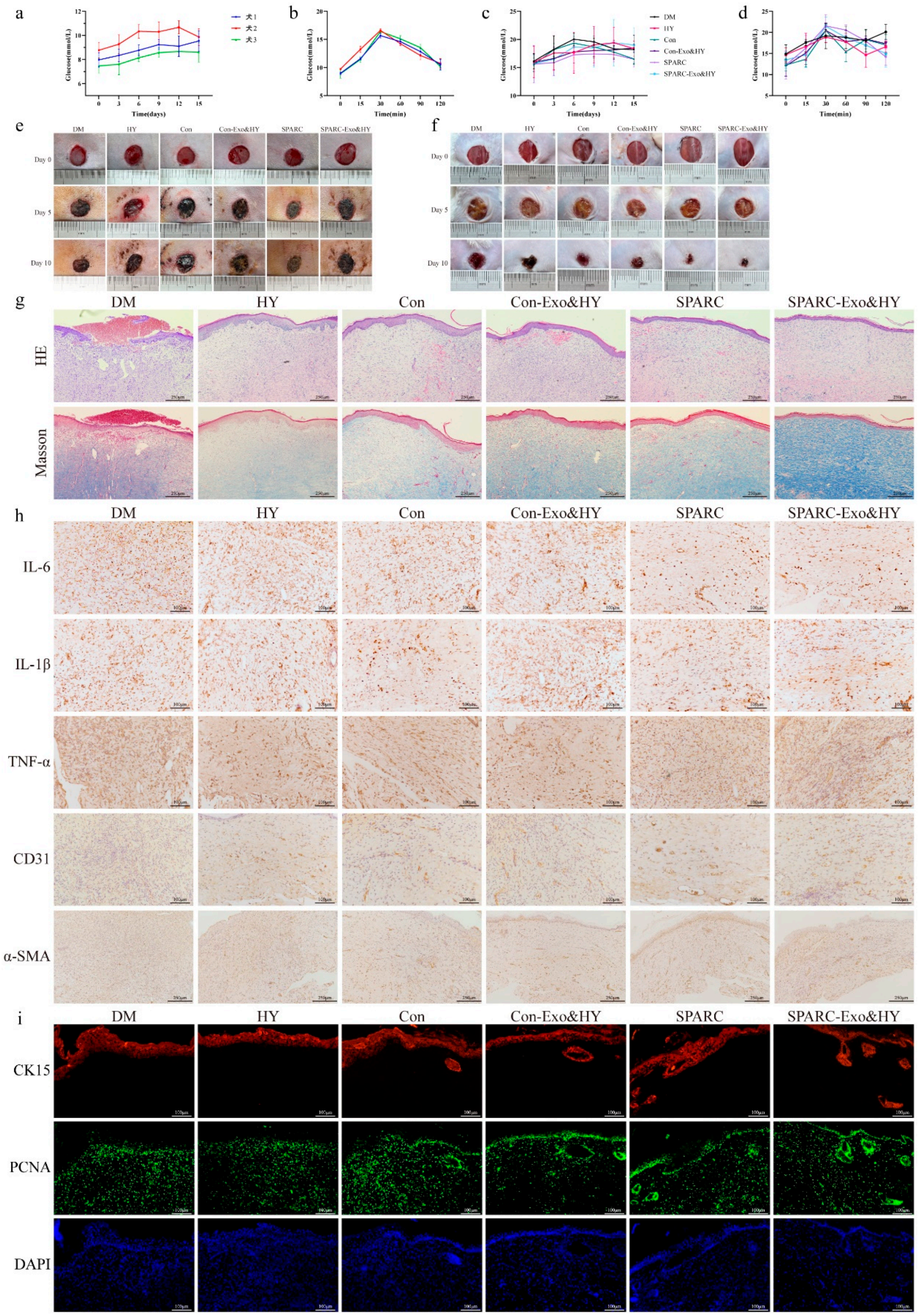

Fig. S3 SPARC-ADSCs promote healing of diabetic skin wound. (a) Diabetic dog blood glucose values; (b) Diabetic dog oral glucose tolerance test (OGTT); (c) Diabetic mouse blood glucose values; (d) Diabetic mouse oral glucose tolerance test (OGTT); (e) Diabetic dog trauma healing macroscopic image; (f) Diabetic mouse trauma healing macroscopic image; (g) Diabetic dog skin tissueparaffin sections stained with hematoxylin and eosin (H&E) and stained with Masson stain; (h) Immunohistochemical staining of paraffin sections of skin tissues of diabetic mice with IL-6,

IL-1β, TNF-α, CD31, α-SMA; (i) Double immunofluorescence staining of paraffin sections of skin tissues of diabetic mice with PCNA and CK15 showing the proliferative state of hair follicle stem cells.

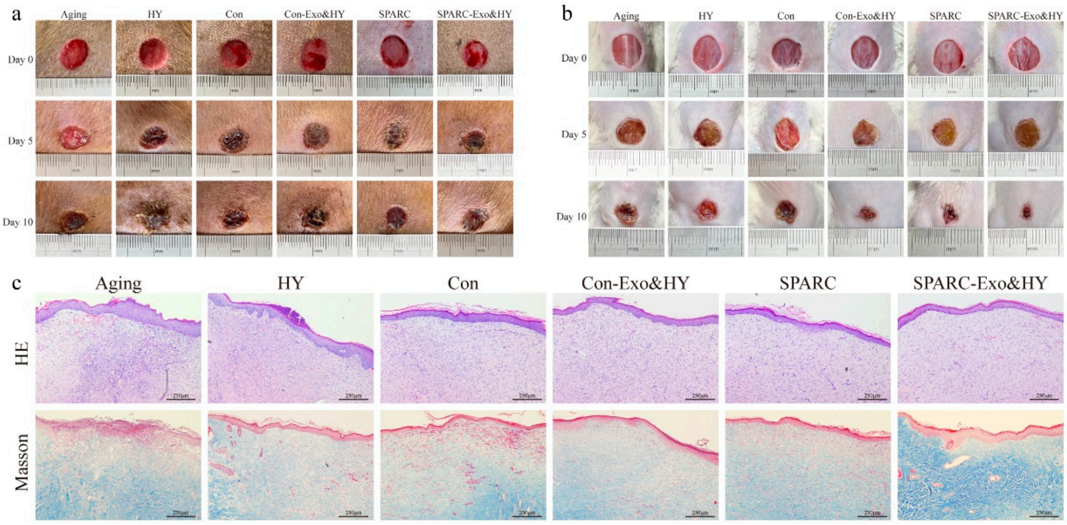

Fig. S4 SPARC-ADSCs promote healing of senescent skin injuries. (a) Gross image of senescent canine trauma healing; (b) Gross image of senescent mouse trauma healing; (c) Hematoxylin and eosin (H&E) staining and Masson staining of senescent canine skin tissues in paraffin sections.
